# Digital Microfluidics for Single Bacteria Capture and Selective Retrieval Using Optical Tweezers

**DOI:** 10.3390/mi11030308

**Published:** 2020-03-15

**Authors:** Phalguni Tewari Kumar, Deborah Decrop, Saba Safdar, Ioannis Passaris, Tadej Kokalj, Robert Puers, Abram Aertsen, Dragana Spasic, Jeroen Lammertyn

**Affiliations:** 1 Department of Biosystems, Biosensors Group, KU Leuven, 3001 Leuven, Belgium; phalgunitewari@gmail.com (P.T.K.); deborah.decrop@gmail.com (D.D.); saba.safdar@kuleuven.be (S.S.); tadej.kokalj@imt.si (T.K.); dragana.spasic@kuleuven.be (D.S.); 2 Department of Microbial and Molecular Systems, KU Leuven, 3001 Leuven, Belgium; ioannis.passaris@gmail.com (I.P.); abram.aertsen@kuleuven.be (A.A.); 3 ESAT-MICAS, Microelectronics and Sensors, KU Leuven, 3001 Leuven, Belgium; robert.puers@kuleuven.be

**Keywords:** optical tweezers, single-cell, digital microfluidics, *Salmonella* Typhimurium

## Abstract

When screening microbial populations or consortia for interesting cells, their selective retrieval for further study can be of great interest. To this end, traditional fluorescence activated cell sorting (FACS) and optical tweezers (OT) enabled methods have typically been used. However, the former, although allowing cell sorting, fails to track dynamic cell behavior, while the latter has been limited to complex channel-based microfluidic platforms. In this study, digital microfluidics (DMF) was integrated with OT for selective trapping, relocation, and further proliferation of single bacterial cells, while offering continuous imaging of cells to evaluate dynamic cell behavior. To enable this, magnetic beads coated with *Salmonella* Typhimurium-targeting antibodies were seeded in the microwell array of the DMF platform, and used to capture single cells of a fluorescent *S.* Typhimurium population. Next, OT were used to select a bead with a bacterium of interest, based on its fluorescent expression, and to relocate this bead to a different microwell on the same or different array. Using an agar patch affixed on top, the relocated bacterium was subsequently allowed to proliferate. Our OT-integrated DMF platform thus successfully enabled selective trapping, retrieval, relocation, and proliferation of bacteria of interest at single-cell level, thereby enabling their downstream analysis.

## 1. Introduction

Heterogeneity exists at every level in living systems, from whole organisms down to single cells. Between individual cells, heterogeneity occurs at the genetic and molecular level, due to stochastic gene expressions, age of cells, asymmetric partitioning during cell division, and inhomogeneous cellular environments [1,2,3,4,5]. Therefore, each cell is unique in its behavior and reactions. It is thus imperative to understand an organism or population down to the individual cell level, which might be essential to delineate complex processes more efficiently, such as the evolution of cancer [6,7], bacterial population dynamics [8,9,10], effect of environmental stimuli at gene-levels [11,12], and modeling of disease prognosis [13,14].

To more selectively analyze a particular cell of interest from a consortium, its retrieval is pivotal. Various solutions have been proposed to date for enabling this. For instance, commercially available fluorescence activated cell sorting (FACS)-based techniques have found extensive applications in cell analysis, primarily for the identification and sorting of individual cells based on their total fluorescence [15]. In addition, myriad other automated and semi-automated techniques based on surface engineering and microfluidics have been investigated in this context [16,17,18,19,20,21,22]. Merely a few examples of such novel technologies for single-cell analysis are: single-cell chromosomal analysis using micropillars [20], monitoring of bacterial drug efflux activity using a hydrophilic-in-hydrophobic (HIH) micropatterned surfaces [21], real-time analysis of individual erythrocytes [17], and real-time cytokine detection [23].

Another technology that has been successfully used for a variety of biological applications, including trapping and sorting of single cells, is optical tweezers (OT) technology (e.g., cell-fusion [24], red blood cell aggregation [25] and deformability [26], microsurgery to hold fragments of chromosomes [27], cell adhesion [28], and cell viability [29,30]). To demonstrate trapping and sorting of single cells, OT have been integrated with channel-based microfluidic platforms. However, the latter can introduce certain level of complexity for liquid manipulation because of their dependence on complex microchannel networks, pumps, and valves [31,32,33,34,35]. Interestingly, the emerging field of digital microfluidics (DMF) has been explored for achieving high throughput single-cell trapping in microwell arrays in a less complex manner, by manipulating fluid droplets on a planar surface [36,37] using electrowetting on dielectric principle (EWOD). To manipulate single magnetic beads (MBs) seeded in a microwell array, Decrop et al. [38,39] recently integrated OT with a DMF platform. They demonstrated that Brownian motion of seeded MBs was essential to optically manipulate MBs. Ionic strength and non-ionic surfactants were altered in a phosphate buffered saline (PBS) buffer to increase electrostatic repulsion and impart steric stabilization, respectively, which increased Brownian motion of the MBs. However, to the best of our knowledge, the manipulation of single cells, trapped in a microwell array, for enabling controlled single-cell analysis has not yet been reported. 

**Figure 1:**
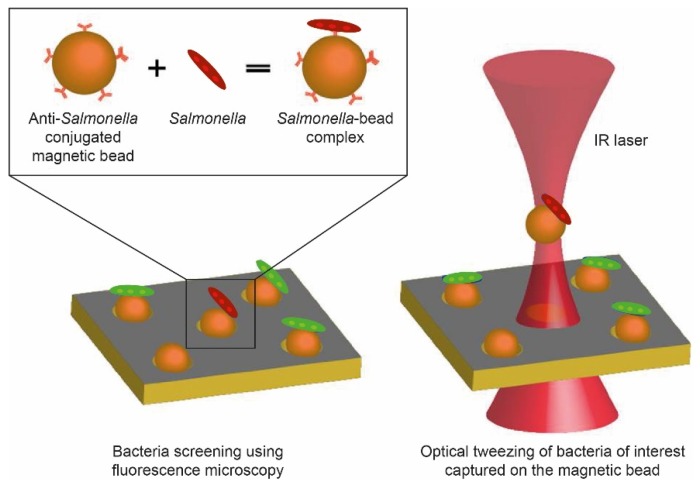
*Salmonella* bacteria bind to MBs conjugated with anti-*Salmonella* antibody, forming thereby *Salmonella*–bead complex, which is subsequently captured using infrared (IR) laser-based OT.

**Figure 2:**
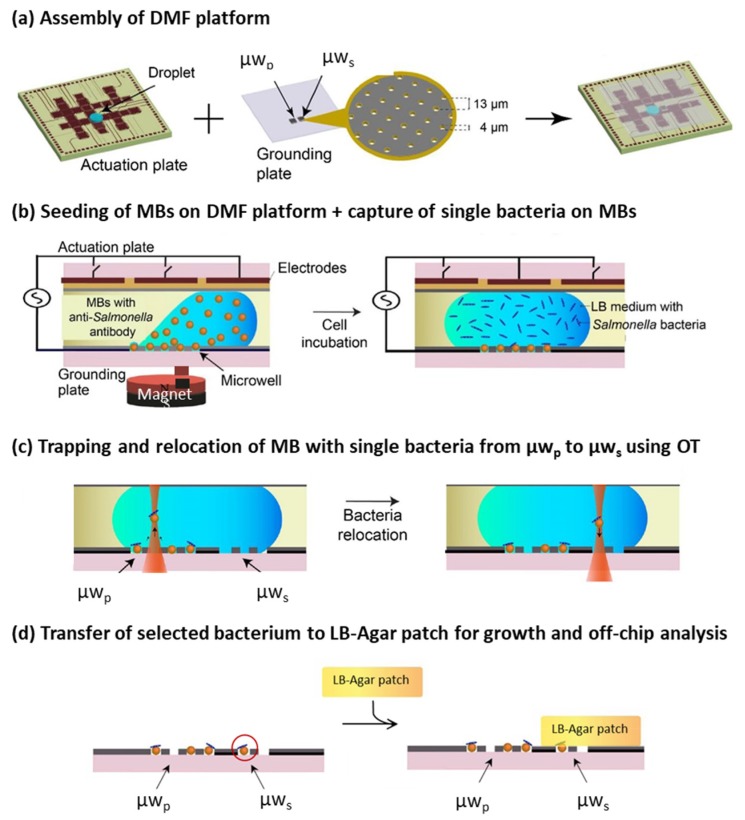
Schematic representation of using the OT-integrated DMF platform for capturing single bacterial cells from microwells, and their subsequent transfer for off-chip analyses. (**a**) Assembly of the DMF platform, comprising an actuation and grounding plate. The grounding plate contains the µw_p_ array and µw_s_ array. (**b**) Manual seeding of MBs (functionalized with anti-*Salmonella* antibody) in the µw_p_ array, and capturing of *Salmonella* on the MBs. (**c**) OT-mediated trapping and relocation of bacteria of interest from µw_p_ to µw_s_, performed after disassembly of the DMF platform by removing the actuation plate. (**d**) LB-agar patch was manually placed on the µw_s_ to allow growth of a colony from the relocated bacterium of interest (red circle).

In this work, we demonstrate integration of OT with a DMF platform for trapping bacteria of interest captured on MBs (Figure 1). Individual cells of *Salmonella enterica* serovar Typhimurium (further referred to as *Salmonella*) were first captured in the microwell array of the DMF platform (Figure 2a) using MBs functionalized with anti-*Salmonella* antibody (Figure 2b, left), forming *Salmonella*–bead complexes (Figure 2b, right). Subsequently, OT were used for selective trapping of a complex in a primary microwell (µw_p_) array (Figure 2c, left) and relocation to a secondary microwell (µw_s_) array (Figure 2c, right). To perform this, cells of interest were selected based on the expression of fluorescent proteins. Finally, the cells were incubated with a lysogeny broth (LB)-Agar patch on top and analyzed for proliferation (Figure 2d). This work thus offers the possibility to spatially organize single bacteria in a 2D format and allows for selecting bacteria at a single-cell resolution, which is challenging with conventional techniques.

## 2. Materials and Methods 

### 2.1. Materials

LB, phosphate buffered saline (PBS), sodium chloride, Tween 40, Tween 60, and Tween 80 were purchased from Sigma Aldrich (Oakville, ON, USA). Fluorinert FC-40 was purchased from 3M (St. Paul, MN, USA) and chemicals for photolithography were supplied by Rohm and Haas (Marlborough, MN, USA). Fluoroalkylsilane Dynasylan F 8263 was supplied by Evonik (Essen, Germany). AZ1505 photoresist and Teflon-AFR were procured from Microchemicals GmbH (Ulm, Germany) and DuPont (Wilmington, DE, USA), respectively. Parylene-C dimer and Silane A174 were purchased from Plasma Parylene Coating Services (Rosenheim, Germany) and superparamagnetic Dynabeads M-280 Tosylactivated were purchased from Thermo Fisher Scientific (Waltham, MA, USA). Ampicillin and isopropyl β-D-1-thiogalactopyranoside (IPTG) were procured from Applichem, Darmstadt, Germany, and L-Arabinose from Acros Organics. Mouse monoclonal anti-*Salmonella* typhimurium LPS antibody (ab8274) was obtained from Abcam (Cambridge, UK).

### 2.2. Digital Microfluidic Platform

The DMF platform consists of: (1) a grounding plate with µw_p_ array and µw_s_ array (at least 1 cm apart) each with 62,500 microwells; and (2) an actuation plate, specifically designed to accommodate and optically visualize both microwell arrays (Figure 2a). Fabrication of the DMF plates was previously described by Witters and co-workers [40,41], and the DMF-operation by Decrop and co-workers [40]. Any adaptations to these are delineated in Section S1. The seeding of MBs, droplet addition, and LB-agar patch attachment and detachment was done manually, while all other manipulations were DMF-mediated.

### 2.3. Cell Culture

*Salmonella enterica* serovar Typhimurium (strain LT2) containing the motility limiting mudY insertion (*Salmonella* Typhimurium LT2 *fliC*::Mu*d*Y) [42,43] was used for microscopy monitoring and manipulation. For conditional expression of mCherry or green fluorescent protein (GFP), the cells were transformed with either pTrc99A-P*_trc_*-mCherry or pFPV-P*_BAD_*-GFP (pAA100 [44]) vector, respectively. The pTrc99A-P*_trc_*-mCherry vector was constructed by ligating an mCherry amplicon into a pTrc99A backbone using EcoRI and BamHI restriction sites. The constructed vector was subsequently verified by both PCR and sequencing (Macrogen, the Netherlands). For culturing of bacteria, LB medium was used either as liquid or solid medium after the addition of 1.52% agar [45]. Stationary phase cultures were obtained by growing *Salmonella* cells overnight for approximately 20 h in LB broth at 37 °C under well aerated conditions (200 rpm on an orbital shaker). Ampicillin (100 µg/mL) and either IPTG (1 mM) or L-arabinose (0.02%) were added to the medium at the indicated final concentrations, for induction of mCherry and GFP expression, respectively.

### 2.4. MB Functionalization

Tosyl-activated MBs (2.5 mg, 83 µL) were washed three times in 500 µL 0.1 M Na-phosphate buffer (pH 7.4). The MBs were then suspended in 75 µL of 0.1 M Na-phosphate buffer (pH 7.4) and 50 µL of 3 M ammonium sulfate buffer (pH 7.4). The suspension was combined with anti-*Salmonella* antibody (final concentration: 0, 0.03, 0.06, 0.12, or 0.24 µg/µL) and incubated with rotation at 1400 rpm for 18 h at 37 °C. After incubation, the MBs were magnetically separated, and the supernatant was discarded. The MBs were resuspended with blocking buffer (0.01 M Na-phosphate buffer with 0.2% BSA, pH 7.4) and incubated on a rotator at 1400 rpm for 2 h at 37 °C. Finally, the MBs were washed 2 times in 500 µL storage buffer (0.01 M Na-phosphate buffer with 0.1% BSA, pH 7.4). The functionalized MBs were resuspended in 120 µL storage buffer (final bead concentration of 4.6 × 10^5^ beads/µL) and stored at 4 °C.

### 2.5. Working Buffer Optimization

For efficient retrieval of MBs using OT, an optimal working buffer composition was required. To achieve that, a panel of different buffer compositions was tested for conducting the initial screening. All buffers were prepared starting from a 1× PBS buffer, varying three parameters: salt concentration (NaCl), surfactant type and surfactant concentration. An I-optimal design of experiments (DOE) was constructed using the JMP software (Pro12, SAS Institute Inc., Cary, NC, USA), where salt and surfactant concentrations were set as continuous variables and surfactant type was taken as a discrete variable [46]. According to the design, 18 feasible buffers were prepared, based on the combinations of the three variables. The detailed buffer compositions are presented in Table S1. For on-chip testing, MBs were washed twice with 200 µL of respective buffer and resuspended at a final concentration of 2.5 × 10^4^ beads/µL for testing on the microwell array. A 10 µL droplet of MB suspension was placed on the microwell array of the grounding plate and manually moved 13–15 times over the microwell array using a pipette tip. A small magnet (NeFeB, 5 mm diameter, 3 mm thickness, 6.86 N, Supermagnete, Gottmadingen, Germany) was placed beneath the microwell array to improve the seeding efficiency. Seeded MBs were then covered with a fresh droplet of the same buffer on the microwell array. The MB vibrations were recorded using a bright field microscope (Eclipse Ti, Nikon, Tokyo, Japan) equipped with a CMOS camera (Zyla 3-tap, Andor, Belfast, UK). The fraction of vibrating MBs from the total seeded MBs was determined and used for analysis. 

### 2.6. Salmonella Capture on Microarray

MBs suspended in the optimal working buffer, at a final concentration of 2.5 × 10^4^ beads/µL, were manually seeded in the µw_p_ array with the aid of a magnet, as indicated before (Figure 2b, left). The droplet containing MBs was replaced by the droplet containing *Salmonella* suspension, with roughly 5 × 10^4^ cells/µL (2.7 µL, 1/20 diluted in working buffer) (Figure 2b, right). Next, the grounding and actuation plates were assembled into the DMF chip and 80 µL silicon oil was added between the plates to avoid evaporation of the droplets. The µw_p_ array was incubated with the *Salmonella* droplet for 30 min before being actuated away from the microwell array. Subsequently, the µw_p_ array was washed twice with the working buffer via DMF-mediated actuation of 2.7 µL droplets (placed on grounding plate prior chip assembly) to remove any nonspecific binding of bacteria. Fluorescence and bright field images (for detecting MBs) were collected using 60× magnification on an inverted fluorescence microscope (Eclipse Ti, Nikon, Tokyo, Japan). Images were processed in ImageJ software (1.47v, NIH, MD) as follows: background subtraction was performed using the rolling ball algorithm with a radius of 50 pixels and salt and pepper noise was removed using the despeckle option. The images were used for counting two events: (i) the number of MBs carrying at least 1 bacteria (at all antibody concentrations); and (ii) the number of bacteria conjugated per MB (only at 0.12 and 0.24 µg/µL antibody concentrations). For the former type of analysis, a grayscale dilation of pixels was performed by applying a maximum filter in the images. In this way, a single bright spot was observed on the MB carrying bacteria, irrespective of the number of bacteria-conjugated to MB. For the latter type of analysis, pixel dilation was not performed and number of bacteria per image were counted manually.

### 2.7. Selective Retrieval of Single Salmonella Bacterium

For selective retrieval of bacteria conjugated to MBs, the previously described OT platform was utilized [38]. In this study, two excitation/emission filters were used for fluorescence applications: FITC ex465-495/em515-555 (for detecting GFP) and TRITC ex540-575/em605-655 (for detecting mCherry). Imaging was done with a Zyla 3-tap CMOS camera (Andor, Belfast, UK). The grounding plate (disassembled from the actuation plate with the magnet still placed underneath), containing both microwell arrays, was positioned on a motorized stage (SCAN IM 120x100, Märzhäuser Wetzlar GmbH & Co.KG, Wetzlar, Germany), facilitating accurate positioning (0.1 µm) of the MBs. Bacteria conjugated to MBs were screened with fluorescence microscopy based on GFP or mCherry fluorescence. Bacteria of interest were held in the optical trap for translocation within the µw_p_ or from the µw_p_ to a µw_s_ (Figure 2c) by repositioning the MB via changes in the x- and y-axis of the microscope stage. To re-seed the trapped bead in the µw_s_, the z-axis of the optical trap was lowered and the aperture of the IR laser was subsequently closed. Following successful repositioning of bacteria, the grounding plate was disconnected from the OT setup and the working buffer on the µw_s_ replaced with a 1 cm × 1 cm LB-agar-ampicillin patch (Figure 2d). The array, with this patch, was incubated at 37 °C to allow bacterial proliferation which was measured after 10 h using fluorescence and brightfield imaging. 

## 3. Results and Discussion

### 3.1. Buffer Selection for OT-Mediated Retrieval of MBs

**Figure 3:**
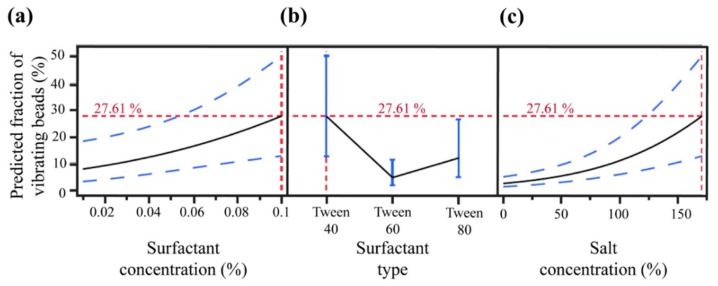
Prediction profiler for evaluating the optimal working buffer composition modeled on: (**a**) surfactant concentration; (**b**) surfactant type; and (**c**) salt concentration. The dotted blue and red lines indicate the confidence interval and maximum predicted fraction of vibrating MBs, respectively.

As previously described by Decrop et al. [38,39], buffer composition is an important aspect for Brownian motion of MBs seeded in microwells, and thus critical for their optical trapping. Therefore, an optimal buffer in this study was selected by performing DOE for investigating salt concentration (NaCl), surfactant type, and surfactant concentration. An I-optimal experimental design was established using the JMP software that prescribed 18 buffer conditions for experimental testing. After determining the fraction of vibrating MBs, the statistical analysis indicated a significant main effect (salt concentration, *p* = 0.0004) and a significant interaction effect (surfactant type × salt concentration, *p* = 0.009), positively influencing the fraction of vibrating MBs. A goodness of fit comparison between predicted and experimental fractions of vibrating MBs resulted in a R^2^ value of 0.88 (Figure S1). To determine the optimal buffer composition, a prediction profiler was generated based on the predicted response and the significant effects. A PBS buffer supplemented with 0.1% (*v*/*v*) Tween 40 and 171 mM NaCl (final NaCl concentration in PBS: 308 mM) was found as the most optimal, with a maximum predicted fraction of 27.6% vibrating MBs (Figure 3). This fraction was not further maximized keeping in view the cytotoxic effect of salt [47,48] and surfactants [49,50]. 

Using the optimal buffer composition, MBs manually seeded in the microwells were retrieved with the OT, achieving a successful retrieval efficiency of 21.3 ± 4.2% (n = 3). This efficiency was slightly lower than the predicted maximum fraction of vibrating beads (27.6%), yet still within the predicted variation interval of (12.6%, 50.1%) (Figure 3). The MBs were observed to be sticking to the microwell as well as escaping the OT trap, possibly due to imbalance of electrostatic charges, resulting in a retrieval efficiency lower than predicted. This may be alleviated by incorporating different chemistries to lower cell sticking to the surface [51]. 

### 3.2. Capturing Single Salmonella Cells on MBs

**Figure 4:**
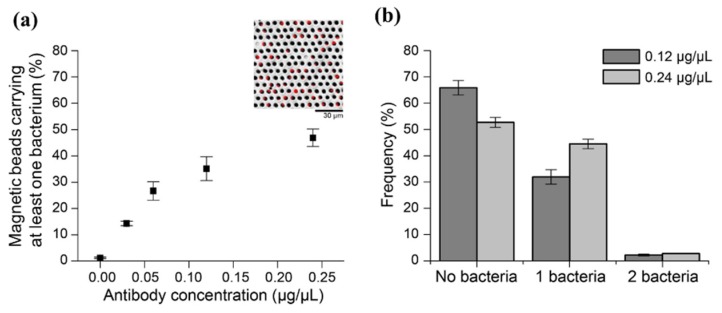
Optimization of immobilized antibody concentration for efficient capture of bacteria on MBs. (**a**) Increase in fraction of MBs carrying at least one bacterium with an increase in antibody concentration can be seen, as analyzed based on fluorescence from mCherry bacteria (**inset**). For each point, a minimum of 11,500 beads were analyzed over three independent repetitions. (**b**) At 0.12 and 0.24 µg/µL of anti-*Salmonella* antibody concentrations, the number of bacteria captured per MB was determined. Error bars represent standard error of the mean of triplicate measurements.

The MBs functionalized with anti-*Salmonella* antibody were manually seeded in microwells, followed by capturing the non-motile strain of *Salmonella* equipped with the mCherry plasmid on the MBs by incubating a droplet containing bacteria over the microwell array. Next, DMF-mediated removal of bacterial droplet and washing of MBs was undertaken. mCherry fluorescence, measured using fluorescence microscopy, was used to calculate the fraction of MBs with captured bacteria. As depicted in Figure 4a, the fraction of MBs with at least one captured bacterium gradually increased from 14% to 47% with increasing antibody concentration used for functionalization of MBs (from 0.0 to 0.24 µg/µL) (Figure S2). Following this preliminary analysis, we further evaluated the number of bacteria captured per MB at the two highest antibody concentrations. For the 0.12 µg/µL antibody concentration, the average fraction of MBs carrying one or two bacteria was 32.8% and 2.3%, respectively, which increased to 44.5% and 2.7% for the 0.24 µg/µL antibody concentration (Figure 4b). Occurrences of single MB carrying two bacteria were observed in both the concentrations. However, such occurrences were significantly lower than the fraction of MB carrying single bacteria (*p* < 0.05). In addition, the difference in the fraction of MBs carrying two bacteria at 0.12 and 0.24 µg/µL antibody concentrations was not statistically significant. Importantly, no MB was observed carrying more than two bacteria for any of the antibody concentrations used. Because the 0.24 µg/µL antibody concentration maximized bacterial capture efficiency, it was implemented for all subsequent OT retrieval and capture experiments. In addition, because no unbound bacteria were observed on the arrays during this work, we concluded that the two washing steps were sufficient for removal of all unbound bacteria. 

**Figure 5:**
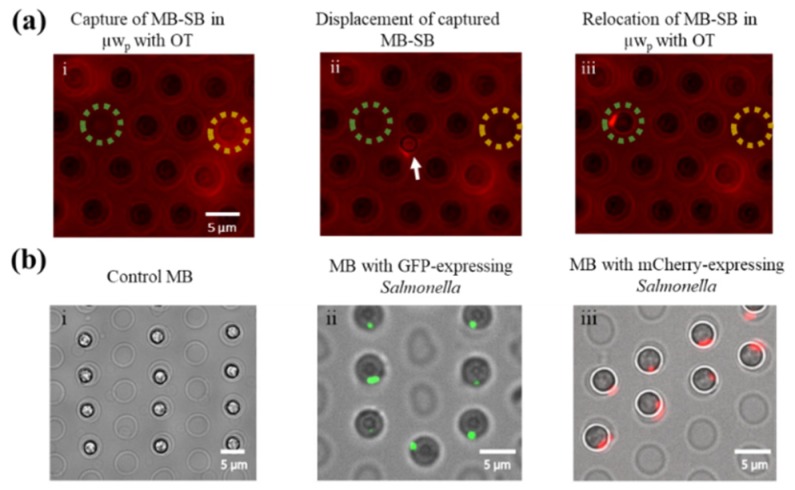
OT-mediated selective retrieval of single bacteria. (**a-i**) MB-SB with mCherry-expression was retrieved from one microwell (yellow circle), (**a-ii**) held in OT-trap, and (**a-iii**) relocated to another microwell (green circle) on the same µw_p_ array, in ~20 s. The OT-held MB-SB during relocation is marked with an arrow in (**a-ii**). (**b-i**) Control MBs, (**b-ii**) MBs with GFP-expressing *Salmonella*, and (**b-iii**) MBs with mCherry-expressing *Salmonella* selectively repositioned using OT.

Following *Salmonella* capture on MBs, the OT were utilized for retrieval of MBs bound with a single bacterium (MB-SB), where the MB acted as the handle for the OT. Figure 5a displays the OT-mediated relocation of a selected MB-SB (with an mCherry) from the yellow dotted circle (Figure 5a-i) to the green dashed circle (Figure 5a-iii) within the same µw_p_ array (images are screenshots of Video S1). The trapping technique was also successfully utilized to spatially arrange individual GFP- or mCherry-expressing bacteria captured on MBs on the microarray, as shown in Figure 5b. Collectively, demonstrated precise control for single-cell manipulation offered by the OT-integrated DMF platform holds great promise for further single-cell sorting and analysis studies.

### 3.3. Growth in Agar Patch

**Figure 6:**
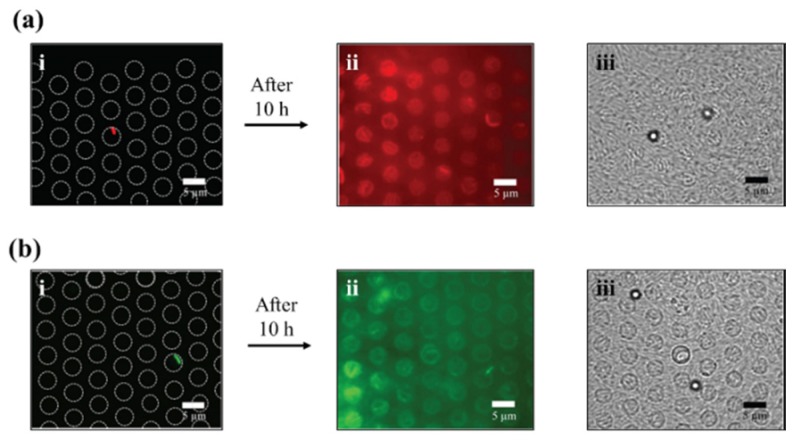
OT-mediated relocation of MB-SB from a µw_P_ to a µw_s_ array for bacteria growth under LB-agar patch. (**a-i**) Fluorescence image of µw_s_ array with mCherry-expressing bacteria on MB at 0 h, (**a-ii**) and at 10 h. (**a-iii**) Brightfield image of µw_s_ array with mCherry-expressing bacteria on MB. (**b-i**) Fluorescence image of µw_s_ array with GFP-expressing bacteria on MB at 0 h, (**b-ii**) and at 10 h. (**b-iii**) Brightfield image of µw_s_ array with GFP-expressing bacteria on MB. Single MB carrying mCherry-expressing (**a**) and GFP-expressing (**b**) bacteria were relocated to a µw_s_ array (**a-i** and **b-i**, respectively), and incubated under a LB-agar patch for proliferation. Fluorescence (ii) and brightfield (iii) images taken after 10 h demonstrated successful, confluent bacterial growth in both the relocated mCherry- and GFP-expressing bacteria. The white dots seen in (**a-iii**) and (**b-iii**) are the MBs, from relocated MB-SB, under brightfield.

The selective retrieval, transfer, and growth of bacteria of interest for off-chip analysis was also realized using the OT setup. An MB of interest, carrying bacteria with mCherry expression (Figure 6a), was successfully transferred from a µw_P_ to a µw_s_ array using OT. The working buffer was replaced with an LB-agar patch (supplemented with arabinose for induction of mCherry expression) (Figure 2d). After allowing bacterial growth at 37 °C for 10 h under the agar patch, imaging revealed that the array retained a fully grown colony, originating from the selected relocated bacteria and infiltrating the entire array (Figure 6a-ii,iii). Here, to avoid optical noise from the overlying bacteria, fluorescence images were focused at the bottom part of the microwell array. In the µw_s_ array, enclosed air bubbles were occasionally observed. Such air bubbles were not observed in the µw_p_ array where MB-SB seeding was performed in the presence of a magnet, as strong magnetic forces pull the MB-SB inside the microwell, thereby displacing the air. The same experiment was performed with MB carrying GFP-expressing bacteria (Figure 6b) using an agar patch supplemented with IPTG, instead of arabinose, for induction of GFP expression.

## 4. Conclusions

The current study demonstrated the ability of the OT-integrated DMF platform for capturing and retrieving bacteria at a single-cell level. To achieve this, the DMF platform consisting of two plates was employed, where microwell arrays were incorporated in the grounding plate. Fluorescence microscopy was utilized to monitor the mCherry and GFP expression continuously during operation: (i) to verify bacterial binding on MBs; (ii) to select MB-SB of interest; and (iii) to validate the proliferation of bacteria. As Brownian motion of MBs is crucial for successful OT [38], we firstly applied DOE for selection of the optimal buffer that allowed effective Brownian motion of MBs used in this study. Next, bacterial capture efficiency of antibody-conjugated MBs was evaluated, and an antibody concentration of 0.24 µg/mL was chosen for MB-functionalization to maximize capture efficiency. Using OT, the MB-SB were captured in microwells and successfully relocated, both on the same array and on different arrays. The former demonstrated controlled, spatial organization of optically retrieved single-cells on the DMF platform, whereas the latter demonstrated successful bacterial proliferation after OT-mediated manipulations, thus indicating bacterial viability. 

In this study, IR OT were used (λ = 1064 nm wavelength, 500 mW) to indirectly manipulate bacteria conjugated to MB, where MB acts as handle [52]. In such cases, the handle can possibly absorb photons from the laser, thereby heating up and transferring the generated heat to the bacteria [53], which can impact cellular responses and thus interfere with analyses. Although our results already suggest that manipulated bacteria can grow and proliferate on the LB-agar patch, future work might focus on testing oscillating OT [54] or combination of different wavelengths/lower power to obviate any ill effects OT treatment that may incur on cellular responses. Alternatively, to reduce photo-damage, an enzymatic scavenging system may be introduced in the medium for removing molecular oxygen [55]. 

In conclusion, this study successfully demonstrated the selective retrieval of single bacteria from a population and presented OT-integrated DMF platform as a tool for manipulating and retrieving single-cells for proliferation, and potential off-chip analysis. This work has brought forth the utility of this coupled technology as a whole, and specifically for its application in library screening and single-cell studies.

## Supplementary Materials

The following are available online at https://www.mdpi.com/2072-666X/11/3/308/s1, Section S1: Fabrication of DMF platform, Table S1: Buffer composition, Figure S1: Goodness of fit plot, Figure S2: Capture of *Salmonella* on MBs, Video S1: Optical tweezing of bacteria.
