# Supplementary material for: Digital Microfluidics for Single Bacteria Capture and Selective Retrieval Using Optical Tweezers
Source: Micromachines (Basel). 2020 Mar 15;11(3):308. doi: 10.3390/mi11030308 (PMC7142809; doi:10.3390/mi11030308)
Supplement: Supplementary file 1 [file micromachines-11-00308-s001.zip › micromachines-745796-proofed/Supplementary Materials.pdf]

micromachines

Supplementary Information: Digital Microfluidics for Single Bacteria Capture and Selective Retrieval Using Optical Tweezers

Phalguni Tewari Kumar, Deborah Decrop, Saba Safdar, Ioannis Passaris, Tadej Kokalj, Robert Puers, Abram Aertsen, Dragana Spasic and Jeroen Lammertyn

S1. Fabrication of DMF Platform

S1.1. Photo-mask

In this work, actuation electrodes of 2.8 × 2.8 mm² were used, each with an inter-electrode gap of 40 µm. For transferring the electrode design on the photo-mask, chrome-on-glass substrates were fabricated by spin-coating a thin layer of AZ® 1505 positive photoresist (Clariant GmbH, Munich, Germany) on chrome coated glass. The mask design was then translated onto a chrome-on-glass substrate using a LASER pattern generator (Microtech LW405, Microtech, Palermo, Italy). The pattern was developed in a basic solution of potassium hydroxide and the exposed chrome was etched using chrome etchant (Cyantek CR-7, Cyantek, Downey, CA, USA).

S1.2. Actuation Plate Fabrication

Actuation plates were fabricated on a high-quality glass wafer (e.g., unpolished Borofloat®, Schott, Mainz, Germany). First, wafers were subjected to thorough cleaning in acetone, isopropyl alcohol (IPA), and de-ionized water for 2 min each. After cleaning, a thin layer of chromium (100 nm) was deposited using a magnetron sputtering unit (Balzers BAE 370, Oerlikon Balzers, Pfäffikon, Switzerland). Next, a thin layer of positive photo-resist (Microposit™ S1818, Rohm and Haas, Bensalem, PA, USA) was spin-coated (10 s, 3000 rpm) on top of the chromium layer and baked for 9 min at 90 °C on a hotplate. Using a contact mask aligner (Karl Suss MJB55 mask aligner, Suss MicroTec, Garching, Germany), the photoresist coated wafer was exposed to UV radiation (50 mJ/cm², 45 s) through the photo-mask and developed in Microposit™ 351-developer (Rohm and Haas, Bensalem, PA, USA), revealing unprotected chromium. Next, chromium etching was performed by submerging the wafer into a bath of Cyantek CR-7 etchant (Cyantek, Downey, CA, USA) (2 min at room temperature). The remaining photo-resist was then stripped from the electrode pattern by immersing the chips in a preheated acetone bath (55 °C) for 15 min followed by a washing step in isopropyl alcohol and water for 2 min each. Next, a thin layer of 3.5 µm Parylene-C, a dielectric material, was deposited on top of the patterned electrode layer using chemical vapor deposition technology (AL 200, Plasma Parylene Systems GmbH, Rosenheim, Germany). Finally, a thin layer (300 nm) of Teflon-AF® 1600 (3 % (w/w) in Fluorinert FC-40) (Dupont, Wilmington, DE, USA) was spin-coated (60 s, 1200 rpm) on top of the Parylene-C layer and baked for 5 min at 110 °C and 10 min at 200 °C.

S1.3. Grounding Plate Fabrication and Micro-patterning

For fabrication of grounding plate, commercially available ITO coated glass plate of size 2.5 × 2.5 cm² were spin-coated with a thin layer (300 nm) of Teflon-AF® 1600 (3 % (w/w) in Fluorinert FC-40) (Dupont, Wilmington, DE, USA) at 1200 rpm for 60 s. Next, these were baked for 5 min at 110 °C and 10 min at 200 °C.

For micro-patterning the hydrophobic layer (Teflon-AF®) of the grounding plate, two mask applications were required. The first mask was important for creating a visualization window in the grounding plate and the second mask was used to pattern the Teflon-AF® layer. To this end, first, glass substrates with a size of 2.2 × 2.2 cm² were prepared (unpolished Borofloat®, Schott, Mainz,

Germany). The glass substrates were thoroughly cleaned in acetone, IPA and de-ionized water for 2 min each. The clean substrates were then covered with a thin layer of aluminum (approximately 40 nm) via thermal evaporation. This layer was semi-opaque, and made optical visualization challenging. Hence, a visualization window on the aluminum layer was fabricated using a mask. To this end, AZ® 1505 positive photo-resist (Clariant GmbH, Oberhausen, Germany) was spin-coated (10 s, 3000 rpm) on top of the aluminum layer and baked for 2 min at 102 °C. The photo-resist was then irradiated with UV (6 mJ/cm^2) through a mask bearing a single square pattern of 2.2 × 2.2 mm^2. Exposed photo-resist was subsequently developed for 45 s in Microposit™ 351-developper (Rohm and Haas, Bensalem, PA, USA). Next, the pattern created in the photo-resist was transferred to the underlying aluminum layer by etching with Transene type A aluminum etchant (Transene Company Inc., Danvers, MA, USA) for 90 s, leaving a small window of 2.2 × 2.2 mm^2 free of aluminum in the center of the grounding plate. The small window was required to allow optical detection on the microwell arrays that were fabricated on the same window on the grounding plate. After aluminum coating, the plate was cleaned in acetone, IPA, and water. As Teflon-AF® adhesion with untreated glass is poor, it was crucial to promote adhesion between Teflon-AF® and glass window using fluorosilane. Hence, adhesion-promoter fluoroalkylsilane Dynasylan® F8263 (Evonik Industries, Essen, Germany) was spin-coated on aluminum-coated glass at 3000 rpm for 10 s, followed by a rinse with pure IPA and baking for 10 min at 100 °C. Next, aluminum-coated plates were spin-coated (60 s, 500 rpm) with a 3 μm thick layer of Teflon-AF® 1600 (6 % (w/w) in Fluorinert FC-40) (Dupont, Wilmington, DE, USA) and baked for 5 min at 110 °C and 10 min at 250 °C.

For micropatterning Teflon-AF® layer, an aluminum hard mask was constructed in the following layers. First, a layer of Parylene-C (approximately 1 μm) was coated on top of the Teflon-AF® by chemical vapor deposition (AL 200, Plasma Parylene Systems GmbH, Rosenheim, Germany). After activating the Parylene-C with a short oxygen-plasma treatment (100 mtorr, 60 W) to improve adhesion, a thin aluminum layer (~100 nm) was deposited by thermal evaporation. Next, AZ® 1505 positive photo-resist (Clariant GmbH, Oberhausen, Germany) was spin-coated (10 s, 3000 rpm) on top of the aluminum layer and baked for 2 min at 102 °C. The photo-resist was then irradiated with UV (6 mJ/cm^2) through the mask bearing the micropattern. Exposed photo-resist was subsequently developed for 45 s in Microposit™ 351-developper (Rohm and Haas, Bensalem, PA, USA). Next, the pattern created in the photo-resist was transferred to the underlying aluminum layer by etching with Transene type A aluminum etchant (Transene Company Inc., Danvers, MA, USA) for 90 s. The pattern was then transferred to the Teflon-AF® layer by dry etching the Parylene-C and Teflon-AF® layers (oxygen-plasma reactive ion etching, 100 W, 150 mtorr) until the glass bottom was reached. Finally, the aluminum mask was removed from the Teflon-AF® by a simple peel-off technique using tweezers [58]. This fabrication process has been used for micro-patterning Teflon-AF® layer with an array consisting of 62,500 microwells of 4 μm diameter and 13 μm depth, aligned above the visualization window in the aluminum.

S1.4. DMF Platform Assembly

In a covered electrowetting on dielectric (EWOD) configuration, droplets were sandwiched and manipulated between an actuation and a grounding plate. In the grounding plate, an electrically conductive path was manually created between the grounding electrode and the external grounding probe by applying a silver conducting paint on one corner of the grounding. The actuation plate was processed further by removing the Parylene-C and Teflon-AF® layer deposited on the contacting pads of the electrodes by manually removing it using a pair of tweezers. To allow space between actuation and grounding plate, double-sided transparent tape (3M, St. Paul, MN, USA) was placed on opposite edges of the actuation plate. Subsequently, droplets were dispensed on the actuation plate by manual pipetting, and the system closed with the grounding plate placed on the spacing tape.

S1.5. Electronic Interface and Software

To manipulate droplets on the DMF platform, the user interface software and an electronic interface were custom made. The interface software enabled the user to define the droplet manipulations to be executed on the DMF chip. This package was composed of two programs: a code generator and a code transformer. The code generator was developed in MATLAB (MathWorks Inc, Natick, MA, USA) for prompting the sequence of droplet manipulations. This sequence was then transmitted to the code transformer, developed in LabView (National Instruments Corp., Austin, TX, USA). Next, the output signals were converted into a transistor–transistor logic (TTL) signal and sent to the electronic interface. Two components were part of the electronic interface: (i) a relay board (designed in-house and fabricated by Eurocircuits, Mechelen, Belgium); and (ii) a chip-holder (designed in-house and fabricated by Verbeeken Precisiemechaniek, Oostkamp, Belgium). The relay board was connected to the computer and received the TTL signals generated by the software, which were then transformed by the relay board to controlled voltage pulses (supplied by an external AC voltage source, 1 kHz sinusoidal signal, 130 VRMS) sent to the chip-holder. The chip-holder contains two units: (i) a base unit used to hold the DMF chip in position; and (ii) a connective unit connected to the relay board to pass the generated voltage pulses to the electrodes of the actuation plate. To this end, spring-loaded pins were provided at the bottom of the connective unit that touch the contact pads of the actuation plate when both units of the chip-holder were brought together. For providing grounding potential, a grounded probe was placed on the silver painted corner of the grounding plate.

Table S1. Buffer composition. Composition of the 18 buffers tested based on I-optimal design of experiments and the resultant fraction of vibrating MBs acquired in each buffer.

Surfactant type	Surfactant concentration (%)	Salt concentration (mM)	Y Vibrating MBs (%)	Predicted Y (%)
Tween 60	0.01	171	4.69	4.71
Tween 40	0.1	0	1.64	1.69
Tween 60	0.1	171	3.09	2.61
Tween 60	0.01	0	4.76	4.53
Tween 60	0.1	0	4.46	5.16
Tween 60	0.01	0	4.97	4.53
Tween 80	0.1	0	1.88	1.50
Tween 40	0.01	0	0.92	1.86
Tween 80	0.01	171	4.87	9.58
Tween 80	0.01	171	7.55	9.58
Tween 80	0.01	0	2.26	1.53
Tween 60	0.01	171	4.69	4.71
Tween 40	0.01	171	6.63	11.14
Tween 80	0.1	0	0.81	1.50
Tween 40	0.1	171	31.22	58.23
Tween 80	0.1	171	14.61	30.69
Tween 40	0.01	0	2.20	1.86
Tween 40	0.1	0	2.96	1.69

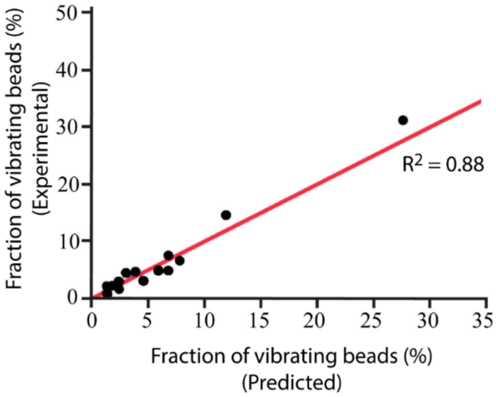

Figure S1. Goodness of fit plot. Plot expressing the goodness of fit of the experimental versus predicted fraction of vibrating MBs obtained in the 18 buffer compositions. The red diagonal line represents the least squares line.

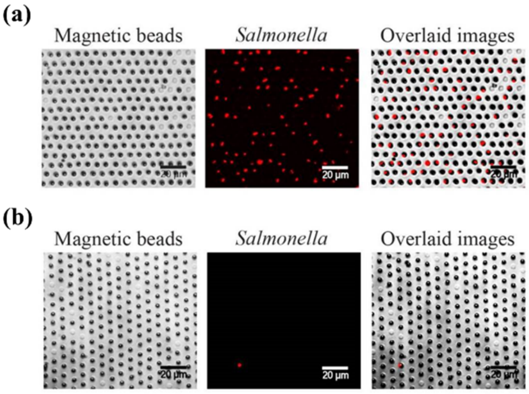

Figure S2. Capture of *Salmonella* on MBs. Images depicting anti-*Salmonella* antibody-functionalized MBs (black rings, leftmost images) capturing bacteria (red fluorescence, middle images) at antibody concentrations of: (**a**) 0.24 µg/µL; and (**b**) 0.0 µg/µL. The right most image is an overlay image of the first two.
